# Increasing incidence of colorectal cancer in young adults in Europe over the last 25 years

**DOI:** 10.1136/gutjnl-2018-317592

**Published:** 2019-05-16

**Authors:** Fanny ER Vuik, Stella AV Nieuwenburg, Marc Bardou, Iris Lansdorp-Vogelaar, Mário Dinis-Ribeiro, Maria J Bento, Vesna Zadnik, María Pellisé, Laura Esteban, Michal F Kaminski, Stepan Suchanek, Ondřej Ngo, Ondřej Májek, Marcis Leja, Ernst J Kuipers, Manon CW Spaander

**Affiliations:** 1 Gastroenterology and Hepatology, Erasmus MC University Medical Center, Rotterdam, The Netherlands; 2 Public Health, Erasmus MC University Medical Center, Rotterdam, The Netherlands; 3 Centre d’investigations Clinique INSERM 1432, CHU Dijon-Bourgogne, Dijon, France; 4 Gastroenterology, Portuguese Oncology Institute of Porto, Porto, Portugal; 5 CINTESIS, Porto Faculty of Medicine, University of Porto, Porto, Portugal; 6 North Region Cancer Registry (RORENO), Department of Epidemiology, Portuguese Oncology Institute of Porto, Porto, Portugal; 7 Epidemiology and Cancer Registry, Institute of Oncology, Ljubljana, Slovenia; 8 Gastroenterology Department, Hospital Clínic de Barcelona, Centro de Investigación Biomédica en Red de Enfermedades Hepáticas y Digestivas (CIBERehd), Institut d’Investigacions Biomediques August Pi i Sunyer (IDIBAPS), Universitat de Barcelona, Barcelona, Spain; 9 Catalan Cancer Plan, Catalan Institute of Oncology, L’Hospitalet del Llobregat, Barcelona, Spain; 10 Cancer Prevention, The Maria Sklodowska-Curie Memorial Cancer Center and Institute of Oncology, Warsaw, Poland; 11 Gastroenterology, Hepatology and Clinical Oncology, Medical Centre for Postgraduate Education, Warsaw, Poland; 12 Department of Health Management and Health Economics, University of Oslo, Oslo, Norway; 13 Internal Medicine, 1st Faculty of Medicine, Charles University, Military University Hospital, Prague, Czech Republic; 14 Faculty of Medicine, Masaryk University, Institute of Biostatistics and Analyses, Brno, Czech Republic; 15 Institute of Health Information and Statistics of the Czech Republic, Prague, Czech Republic; 16 Institute of Clinical and Preventive Medicine & Faculty of Medicine, University of Latvia, Riga, Latvia

**Keywords:** colorectal cancer, epidemiology, screening

## Abstract

**Objective:**

The incidence of colorectal cancer (CRC) declines among subjects aged 50 years and above. An opposite trend appears among younger adults. In Europe, data on CRC incidence among younger adults are lacking. We therefore aimed to analyse European trends in CRC incidence and mortality in subjects younger than 50 years.

**Design:**

Data on age-related CRC incidence and mortality between 1990 and 2016 were retrieved from national and regional cancer registries. Trends were analysed by Joinpoint regression and expressed as annual percent change.

**Results:**

We retrieved data on 143.7 million people aged 20–49 years from 20 European countries. Of them, 187 918 (0.13%) were diagnosed with CRC. On average, CRC incidence increased with 7.9% per year among subjects aged 20–29 years from 2004 to 2016. The increase in the age group of 30–39 years was 4.9% per year from 2005 to 2016, the increase in the age group of 40–49 years was 1.6% per year from 2004 to 2016. This increase started earliest in subjects aged 20–29 years, and 10–20 years later in those aged 30–39 and 40–49 years. This is consistent with an age-cohort phenomenon. Although in most European countries the CRC incidence had risen, some heterogeneity was found between countries. CRC mortality did not significantly change among the youngest adults, but decreased with 1.1%per year between 1990 and 2016 and 2.4% per year between 1990 and 2009 among those aged 30–39 years and 40–49 years, respectively.

**Conclusion:**

CRC incidence rises among young adults in Europe. The cause for this trend needs to be elucidated. Clinicians should be aware of this trend. If the trend continues, screening guidelines may need to be reconsidered.

**Significance of this study**

**What is already known on this subject?**

- An increase in the incidence of colorectal cancer (CRC) among subjects aged 20–40 years has been observed in North America, Australia and China.
- The American Cancer Society therefore recently recommended to lower the age to start screening from 50 to 45 years.

**What are the new findings?**

- The incidence of CRC increases in Europe among subjects aged 20–49 years.
- The fastest rise in incidence occurs in the youngest age group.
- The rise in incidence was more prominent for colon cancer than for rectal cancer.
- The rise in incidence is not associated with a similar rise in mortality.

**How might it impact on clinical practice in the foreseeable future?**

- The increasing incidence of CRC among younger subjects asks for reconsideration of screening guidelines.
- A continued increase in incidence will require to lower the age to start screening.
- Further research is needed to identify underlying aetiological factors.
- Clinicians should be aware of the rising incidence of CRC in young adults.

## Introduction

The overall crude incidence of colorectal cancer (CRC) increased in most European countries over the last decade. The annual increase ranged in different countries between 0.4% and 3.6%.^1^ The recent introduction of CRC screening in most European countries will likely reverse this trend.^2 3^ These screening programmes typically target subjects aged 50 years and above. In several parts of the world, the CRC incidence has also risen in individuals below 50 years of age. In the USA, the incidence of colon cancer increased since 1974 with 1.0%–2.4% annually and the incidence of rectal cancer with 3.2%.^4^

The possible reasons for this increasing incidence are unknown, but may be related to the increasing prevalence of obesity, lack of exercise and to dietary factors such as alcohol and processed meat.^3^ Furthermore, urbanisation and pollution have been implicated in the overall increase in cancer incidence.^5^ CRC in young adults is in part due to hereditary cancer syndromes, but most cases are sporadic.^6^

The changing epidemiology of CRC may also have practical implications, in particular for age to start screening. With the use of the Microsimulation Screening Analysis simulation model, we previously showed that screening initiation at age 45 years had in the US population a favourable balance between screening benefits and burdens.^7^ This finding supported the American Cancer Society to recommend starting screening at age 45 years instead of 50 years.^8^

Whether the incidence of CRC also increases among young adults in Europe has not been investigated. We therefore analysed trends in CRC incidence in this population.

## Methods

### Study design and data source

Data on age-specific incidence and mortality of CRC by year of diagnosis were retrieved from national and regional European cancer registries with a time frame of at least 10 years (online supplementary table 1). We evaluated incidence and mortality of CRC, colon cancer (ICD-O-3 codes C18) and rectal cancer (C20) between 1990 and 2016. Data were collected for subjects aged 20–49 years. Five-year incidence and mortality rates were collected and expressed per 100 000 persons.

10.1136/gutjnl-2018-317592.supp1 Supplementary file 1

### Statistical analysis

Temporal trends in CRC incidence within the study period were investigated using Joinpoint regression analyses, applying an algorithm to define significant changes in temporal trends on a logarithmic scale. The annual percent change (APC) in each Joinpoint segment represents the rate of change in cancer incidence per year in a given time period. The analyses were performed using the Joinpoint Regression Programme 4.5.0.1, National Cancer Institute.

All tests of statistical significance were two-sided; a p value of <0.05 was considered significant. Incidence rates were calculated for three age groups (20–29, 30–39, 40–49 years), presented per 100 000 persons and adjusted to population numbers for each country.

As not all countries could provide data over the entire time period, a sensitivity analysis was performed with data from countries that covered the entire time frame.

We set out to distinguish between a period effect and a cohort effect. While a period effect results from external factors that equally affect all age groups at a particular time period, a cohort effect represents variations resulting from unique exposure of a specific birth cohort. To this aim, we identified for each age group the year in which the increase in CRC incidence, if any, had started. If it were to be the same for the three age groups, the increase in incidence was considered to be a period effect. If the starting year were to be more recent in the older age groups, the increase was considered to be a cohort effect.

## Results

**Figure 1:**
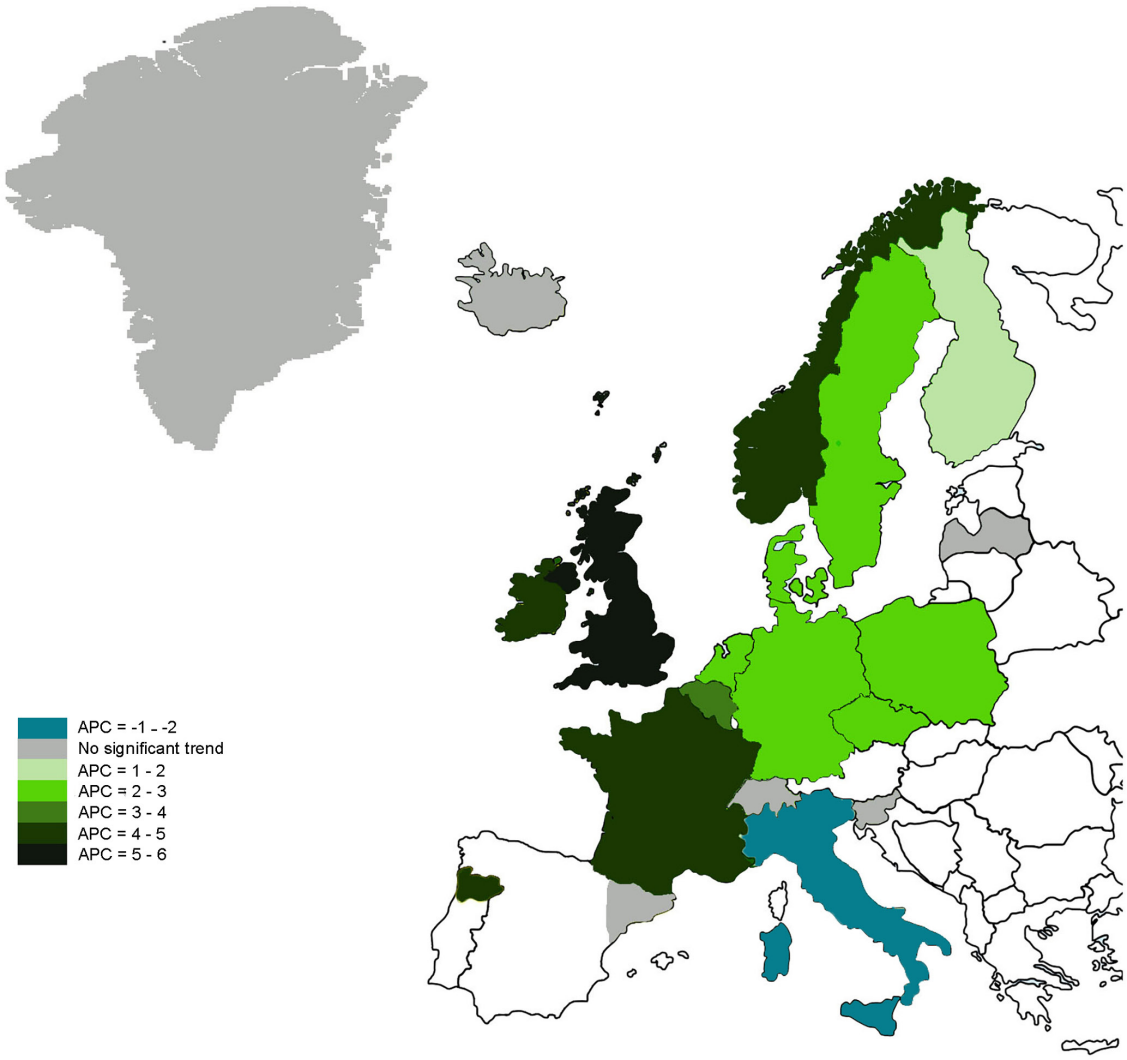
Annual percent change (APC) in colorectal cancer (CRC) incidence from the European countries included in the analysis in adults aged 20–39 years, 1990–2016. Light green to dark green: significant increase in CRC incidence rate; blue: significant decrease in CRC incidence rate; grey: no significant trend.

Incidence data were available from 20 European countries (figure 1); mortality data from 16 of those (not including Belgium, France, the UK and Ireland). In 2009, the population of these 20 countries numbered 91 842 346 individuals aged 20–39 years, of whom 47 364 were diagnosed with CRC from 1990 to 2016, and 51 868 457 individuals aged 40–49 years, of whom 140 554 were diagnosed with CRC from 1990 to 2016.

### Incidence of colorectal cancer

#### Age group 20–29 years

**Figure 2:**
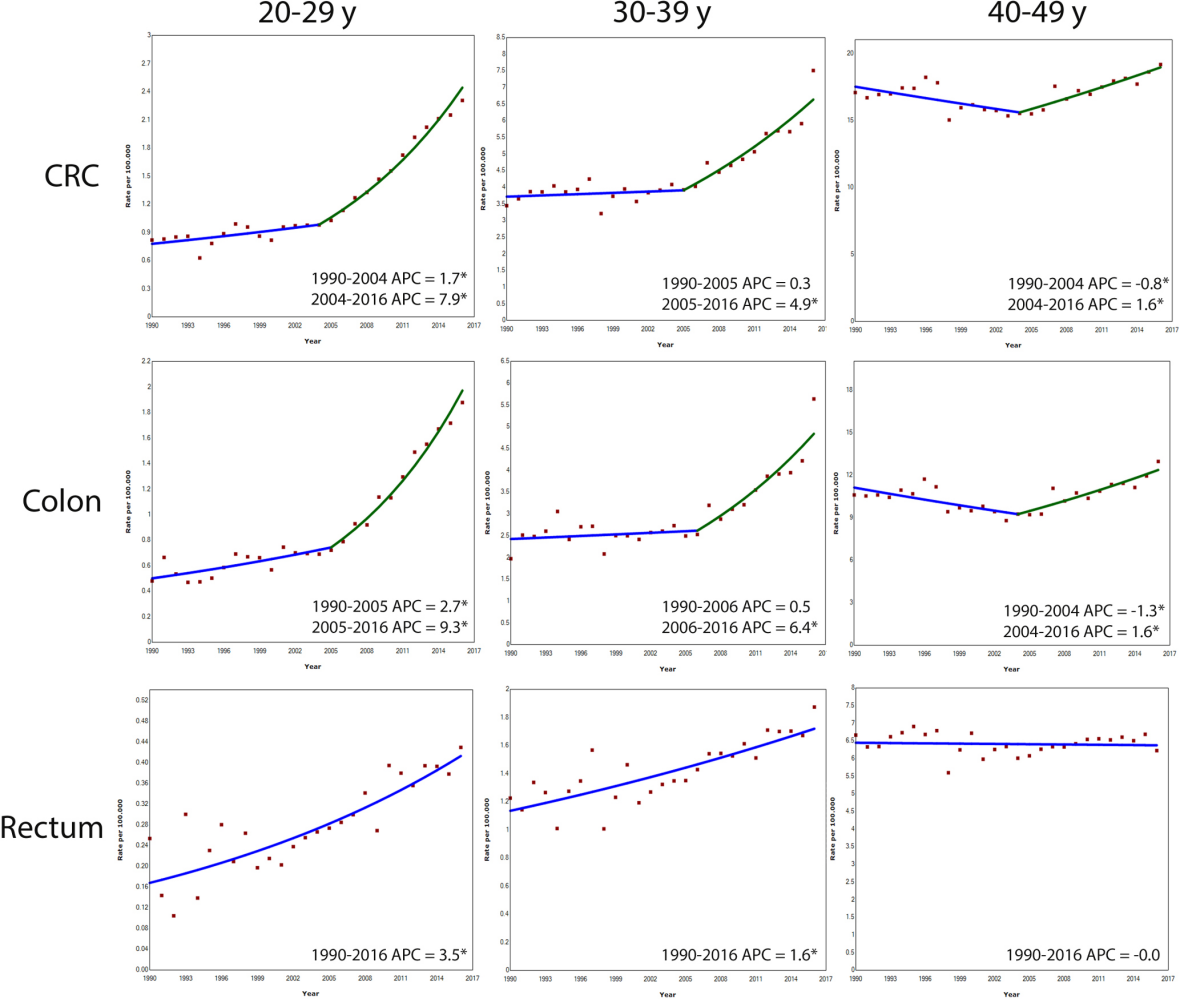
Annual percent change (APC) in age-specific colorectal cancer (CRC), colon cancer and rectal cancer incidence rates in Europe, 1990–2016. *Indicates that APC is statistically significant different from zero.

For both sexes combined, CRC incidence increased from 0.8 to 2.3 cases per 100 000 persons between 1990 and 2016. This increase was 1.7% per year between 1990 and 2004, and then rose to 7.9% increase per year between 2004 and 2016 (figure 2). In men, the CRC incidence increased with 2.6% per year between 1992 and 2005. This increase rose to 7.4% per year between 2005 and 2016. In women, the CRC incidence increased with 1.8% per year between 1990 and 2003 and with 8.1% per year between 2003 and 2016. The incidence of colon cancer rose more markedly (2.7% per year between 1990 and 2005 and 9.3% per year between 2005 and 2016) than the incidence of rectal cancer. The latter increased with 3.5% annually throughout the whole period without an acceleration over time.

#### Age group 30–39 years

For both sexes combined, in age group 30–39 years the CRC incidence increased, although less steeply than in age group 20–29 years (figure 2). In men, the CRC incidence increased with 3.4% per year between 2001 and 2016 (from 3.7 to 7.1 cases per 100 000 persons between 1990 and 2016). In women, no significant change in trend was observed between 1990 and 2005, but the CRC incidence increased with 6.8% annually between 2005 and 2016 (from 2.8 to 6.4 cases per 100 000 persons between 2006 and 2016). The colon cancer incidence increased between 2006 and 2016 with 6.4% per year; that of rectal cancer with 1.6% per year between 1990 and 2016.

#### Age group 40–49 years

In age group 40–49 years, the CRC incidence decreased with 0.8% between 1990 and 2004, but increased with 1.6% per year between 2004 and 2016 (incidence increased from 15.5 to 19.2 cases per 100 000 persons between 2005 and 2016). The same trend was observed for colon cancer: the incidence decreased with 1.3% per year between 1990 and 2004 and then increased with 1.6% annually between 2004 and 2016. No significant change in trend was observed for rectal cancer (figure 2).

#### Country-specific trends

Trends in incidence of CRC per European region are shown in figure 1. CRC incidence increased significantly among subjects aged 20–39 years in 12 countries: Belgium, Germany, the Netherlands, the UK, Norway, Sweden, Finland, Ireland, France, Denmark, Czech Republic and Poland. Italy showed a decrease in incidence in this age group. No significant change was observed in the remaining six countries (online supplementary figure 1).

CRC incidence increased significantly among subjects aged 40–49 years in eight countries: the UK, Greenland, Sweden, Slovenia, Germany, Finland, Denmark and the Netherlands. Only Czech Republic showed a significant decrease in incidence from 1997 to 2015. No significant change was observed in the remaining 11 countries (online supplementary figure 2).

#### Sensitivity analyses

**Figure 3:**
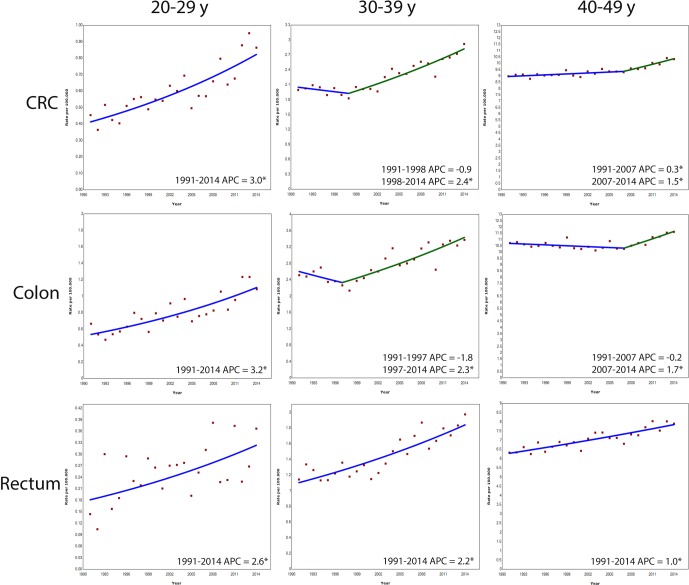
Annual percent change (APC) in age-specific colorectal cancer (CRC), colon cancer and rectal cancer incidence rates in nine European countries, 1991–2014. Analyses on trend in incidence of CRC was based on nine countries: Slovenia, Norway, Denmark, Sweden, Finland, the Netherlands, Czech Republic, Switzerland and Greenland. Analyses on trend of incidence of colon cancer and rectum cancer was based on eight countries: Slovenia, Norway, Denmark, Sweden, Finland, the Netherlands, Czech Republic and Greenland. *Indicates that APC is statistically significant different from zero.

Not all countries could provide data over the entire time period of 1990 to 2016. We therefore performed sensitivity analyses for the longest possible time frame: 1991 to 2014. Data from nine countries were included: Denmark, Finland, Norway, Sweden, the Netherlands, Greenland, Slovenia, Czech Republic and Switzerland. The outcomes indicated increases in the incidence of both colon and rectal cancer in all age groups (figure 3).

#### Period or cohort effect

We assessed by means of sensitivity analysis whether the increase in incidence was a period or a cohort effect (figure 3). This showed that adults aged 20–29 years had an increase in CRC incidence from 1991 to 2014. In age group 30–39 years, a rise in incidence started in 1998 and exactly 10 years later (2007) a rise in incidence was observed among those aged 40–49 years. This difference in starting points is compatible with a cohort effect.

### Mortality due to colorectal cancer

#### Age group 20–39 years

**Figure 4:**
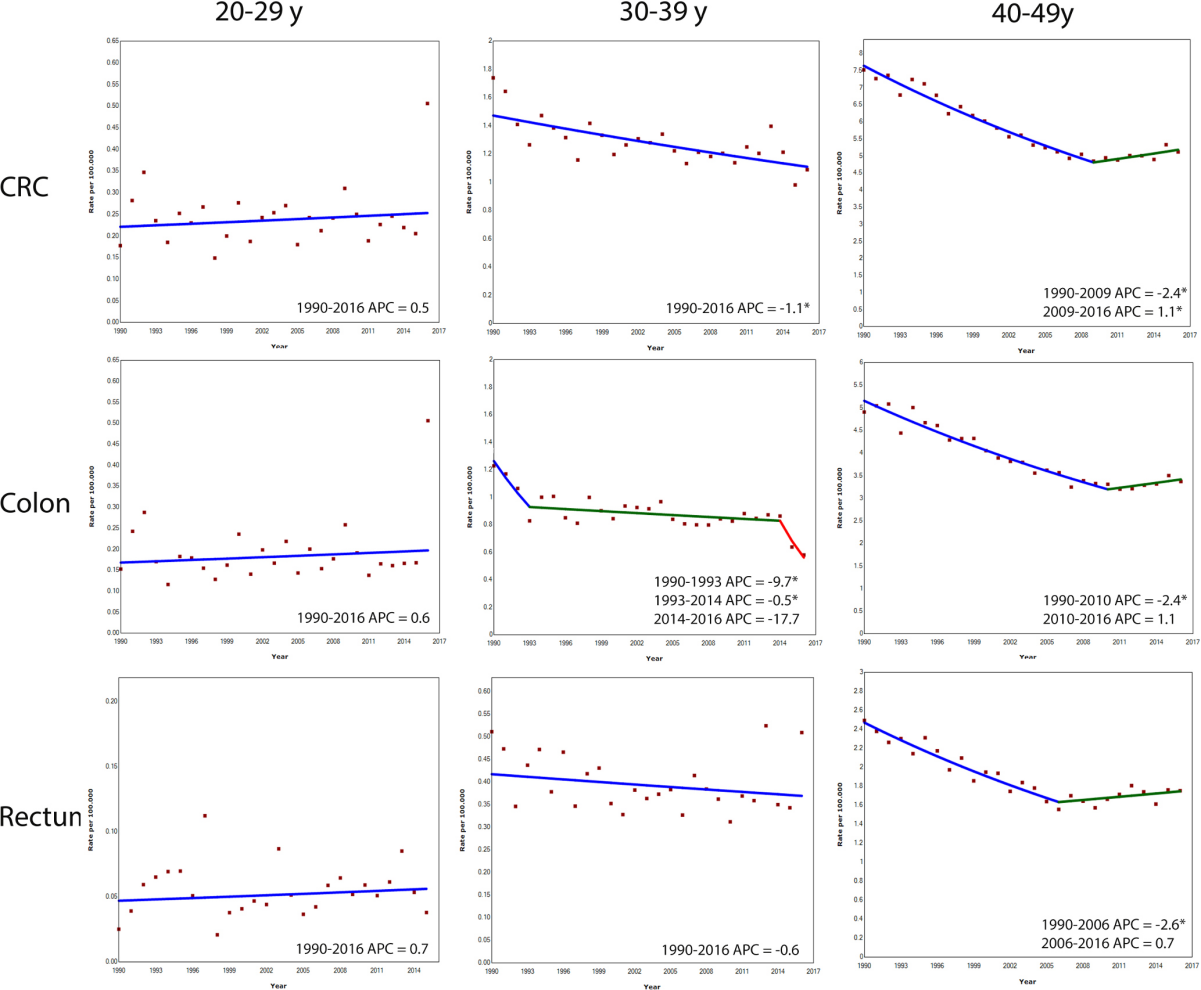
Annual percent change (APC) in age-specific colorectal cancer (CRC), colon cancer and rectal cancer mortality rates in Europe, 1990–2016. *Indicates that APC is statistically significant different from zero.

The mortality rate for CRC did not significantly change in the age group 20–29 years. In the age group 30–39 years, the mortality decreased with 1.1% per year (figure 4). The mortality rate of colon cancer decreased with 9.7% per year between 1990 and 1993, and with 0.5% per year between 1993 and 2014, to remained stable from 2014 onwards. No significant change in mortality was observed for rectal cancer.

#### Age group 40–49 years

The overall mortality of CRC in the age group 40–49 years decreased with 2.4% per year between 1990 and 2009, but increased with 1.1% per year between 2009 and 2016 (figure 4). The mortality rate of colon cancer decreased with 2.4% per year between 1990 and 2010, and remained stable between 2010 and 2016. The mortality rate of rectal cancer decreased with 2.6% per year between 1990 and 2006, and remained stable between 2006 and 2016.

## Discussion

Our study showed an increase in CRC incidence in adults aged 20–49 years in Europe. The largest increase in CRC incidence occurred among subjects aged 20–39 years. The incidence of colon cancer increased with 6.4%–9.3% annually; that of rectal cancer with 1.6%–3.5% per year. The causes of this increase are yet unknown. Awareness of this trend is relevant to identify patients at risk. Further research is needed to determine whether the trend can be reversed, among others by lowering the age to start screening.

In the past years, an increase in CRC incidence in young adults has been observed in different parts of the world, such as the USA.^4^ In Canadian subjects aged 20–29 years, the incidence of colon cancer rose faster than that of rectal cancer (APC 6.2%, respectively 1.5%). CRC incidence in young adults also rises in Australia and China. In the latter country, adoption of a Western lifestyle is thought to contribute to this trend.^9 10^

In the USA, the increase in CRC incidence was explained by a cohort effect. Our data support a similar effect in Europe. The incidence started to rise exactly 10 years earlier in the age groups 30–39 years than in the group of 40–49 years. CRC incidence also rose among those aged 20–29 years, however, with no turning point during the study period. This suggests that the turning point already occurred before 1990.

The cause of this trend is unknown. A combination of factors is likely to have contributed. This includes the increasing prevalence of obesity. The latter parallels the increase in CRC incidence in young adults.^11^ A meta-analysis showed that weight gain is associated with an increased risk of CRC.^12^ Excess nutrients may initiate a chronic low-grade inflammatory response in metabolic cells.^13^ Also, other risk factors such as lack of physical activity, increased alcohol intake and cigarette smoking may play a role.^14–17^

We found that the rate of increase differed for colon and rectal cancer, ranging from 1.6% to 9.3% for colon cancer vs 0% to 3.5% for rectal cancer. Although the above-mentioned risk factors apply to both colon and rectal cancer, some factors are strongly associated with colon cancer only. Lifestyle factors such as diet, physical activity and alcohol have been associated with risk of colon cancer, but not with rectal cancer.^18^ Also, a meta-analysis showed that obesity was in particular associated with an increased risk of colon cancer. For rectal cancer this association was less apparent in men, and absent in women.^19^ This might in part be explained by the greater susceptibility of the colon to the effects of insulin in comparison with the rectum.^20^

The increasing use of colonoscopy for diagnostic and screening purposes may have been responsible for a proportion of the detected CRCs in young adults. Nevertheless, detection bias is probably not the driving factor for this trend, since young adults are less likely to be screened for CRC, the rise was most marked in the youngest age group and the turning points differed between age groups.

Current guidelines in Europe recommend CRC screening from the age of 50. In 2018, the American Cancer Society recommended to start screening at the age of 45. This recommendation was based on the burden of disease, the increasing incidence among younger subjects, the results of modelling and the assumption that screening the age group 45–49 years will have preventive effect as screening those 50 years and above. The American Cancer Society’s analyses showed a favourable benefit-to-burden balance with an expected reduction in CRC mortality and incidence.^8^ For several reasons, the results of our study provide no argument for starting screening at the age of 45 years in Europe. First, the largest increase in CRC incidence rate was observed in the age group of 20–39 years. Second, the rate of change in CRC incidence differed between countries. Third, the absolute numbers of CRC in these age groups still remain low in comparison with elderly subjects. Fourth, most European countries struggle to find the resources to properly screen the age group of 50–75 years, or are in the process of implementing screening for this group. For these reasons, it is too early to use our data to support screening for those aged 45–50 years. However, it is relevant to research to monitor this trend, and repeatedly assess whether screening practice needs to be adapted. Furthermore, we should find underlying causes, and identify high-risk subjects who might benefit from earlier screening. A first step to reach this goal is to make clinicians aware that the CRC incidence in young adults is rising quite rapidly.

Italy is the only country that showed a significant decrease in CRC incidence among subjects aged 20–39 years. This occurred at a rate of 1.8% per year from 1998 onwards. We should be careful with data interpretation though, because the observation might be due to selection bias. The Italian data were retrieved from the AITRUM database, covering only nine regions from 1996 to 2009 instead of the entire country over a longer period.

The incidence trend did not significantly change in Greenland, Iceland, Slovenia, Catalonia, Latvia and Switzerland. This can likely be explained by the low population numbers in these countries, affecting power of our calculations.

This study is the first to give an overview of CRC incidence and mortality rates in younger adults in Europe. A major strength is the use of data from 20 European counties. Still, several limitations need to be addressed. First, not all European Union member countries could be included, either because of the lack of a national cancer registry or inaccessibility of the data. Also, for some countries (Portugal, Spain and Italy), data were only available for only a limited number of regions. Second, not all countries could provide data over a period of 25 years, because some national cancer registries were set up in a later year. In all countries, however, data were available for at least 10 years. The analysis of data from countries with a longer observation period (1991–2014) consistently showed the same trends. Third, the quality of data differed between countries. Data quality was estimated in terms of microscopically verified (MV) and death certificate only (DCO). The German data, for example, had an MV rate of 85.6% and a DCO rate of 13%. The Latvian data had an MV rate of 80.7% and a DCO rate of 5.5%. Fourth, the national cancer registries from Switzerland and Germany present estimated nationwide data on CRC incidence, because not all regions can provide CRC incidence and mortality rates. Fifth, individual data were not accessible. It was not possible, therefore, to differentiate between left and right colon cancers and pathological characteristics of patients with CRC could not be retrieved.

In conclusion, the incidence of CRC is rising in Europe among subjects aged 20–49 years. If this trend continues, screening guidelines may need to be reconsidered. Until the underlying cause of this trend is clarified, it would be commendable to raise clinicians’ awareness and identify factors possibly associated with this trend.
