# Supplementary material for: Increasing incidence of colorectal cancer in young adults in Europe over the last 25 years
Source: Gut. 2019 May 16;68(10):1820–6. doi: 10.1136/gutjnl-2018-317592 (PMC6839794; doi:10.1136/gutjnl-2018-317592)
Supplement: Supplementary file 1 [file gutjnl-2018-317592supp001.pdf]

Supplementary file belonging to:

Increasing incidence of colorectal cancer in young adults in Europe over the last 25 years

Fanny E.R. Vuik[1]; Stella A.V. Nieuwenburg[1]; Marc Bardou[2,3]; Iris Lansdorp-Vogelaar[2]; Mário Dinis-Ribeiro[4,5]; Maria J. Bento[6]; Vesna Zadnik[7]; Maria Pellisé[8]; Laura Esteban[9]; Michal Kaminski[10,11,12]; Stepan Suchanek[13]; Ondřej Ngo[14,15]; Ondřej Májek[14,15]; Marcis Leja[16]; Ernst J. Kuipers[1]; Manon C.W. Spaander[1]

Country	Incidence	Mortality	Population	Microscopically verified (MV) (%)*	DCO (death certificate only) rate (%)*	Level of completeness°	National coverage°
**The Netherlands**	The Netherlands Cancer Registry, IKNL, 2018.<br><br>https://www.cijfersoverkanker.nl	The Netherlands Cancer Registry, IKNL, 2018.<br><br>https://www.cijfersoverkanker.nl	United states CENSUS bureau<br><br>https://www.census.gov/en.html	97.3	-	100%	All data available from 1989
**Germany**	The German Centre for Cancer Registry Data (ZfKD)<br><br>www.krebsdaten.de/database	The Federal Statistical Office Germany	United states CENSUS bureau<br><br>https://www.census.gov/en.html	85.6	13	90%	All data available from 1999
**Belgium**	The Belgium cancer registry<br><br>http://www.kankerregister.org/Home_en	NA	United states CENSUS bureau<br><br>https://www.census.gov/en.html	99.0	-	-	95%
**Ireland**	National cancer registry Ireland<br><br>https://www.ncri.ie	NA	United states CENSUS bureau<br><br>https://www.census.gov/en.html	93.5	1.0	98.2	All data available from 1994
**Italy**	AIRTUM ITACAN: Cancer in Italy, Version 2.0. Italian Association of Cancer Registries<br><br>http://www.registri-tumori.it	AIRTUM ITACAN: Cancer in Italy, Version 2.0. Italian Association of Cancer Registries<br><br>http://www.registri-tumori.it	United states CENSUS bureau<br><br>https://www.census.gov/en.html	87.9	2.5	-	70% of data available from 2006
**Denmark**	Association of the Nordic Cancer Registries. Danish Cancer Society.	Association of the Nordic Cancer Registries. Danish Cancer Society.	United states CENSUS bureau<br><br>https://www.census.gov/en.html	95.7	0.3	100%	-

	http://www-dep.iarc.fr/NORDCAN/english/frame.asp	http://www-dep.iarc.fr/NORDCAN/english/frame.asp					
**Sweden**	Association of the Nordic Cancer Registries. Danish Cancer Society. http://www-dep.iarc.fr/NORDCAN/english/frame.asp	Association of the Nordic Cancer Registries. Danish Cancer Society. http://www-dep.iarc.fr/NORDCAN/english/frame.asp	United states CENSUS bureau https://www.census.gov/en.html	-	-	100%	All data available from 1960
**Norway**	Association of the Nordic Cancer Registries. Danish Cancer Society. http://www-dep.iarc.fr/NORDCAN/english/frame.asp	Association of the Nordic Cancer Registries. Danish Cancer Society. http://www-dep.iarc.fr/NORDCAN/english/frame.asp	United states CENSUS bureau https://www.census.gov/en.html	94.6	0.8	100%	All data available from 1953
**Finland**	Association of the Nordic Cancer Registries. Danish Cancer Society. http://www-dep.iarc.fr/NORDCAN/english/frame.asp	Association of the Nordic Cancer Registries. Danish Cancer Society. http://www-dep.iarc.fr/NORDCAN/english/frame.asp	United states CENSUS bureau https://www.census.gov/en.html	-	-	100%	All data available from 1953
**Iceland**	Association of the Nordic Cancer Registries. Danish Cancer Society. http://www-dep.iarc.fr/NORDCAN/english/frame.asp	Association of the Nordic Cancer Registries. Danish Cancer Society. http://www-dep.iarc.fr/NORDCAN/english/frame.asp	United states CENSUS bureau https://www.census.gov/en.html	>97.9	<0.3	100%	All data available from 1955

**Greenland**	Association of the Nordic Cancer Registries. Danish Cancer Society.<br><br>http://www-dep.iarc.fr/NORDCAN/english/frame.asp	Association of the Nordic Cancer Registries. Danish Cancer Society.<br><br>http://www-dep.iarc.fr/NORDCAN/english/frame.asp	United states CENSUS bureau<br><br>https://www.census.gov/en.html	-	-	100%	All data available from 1968
**Switzerland**	The Swiss national dataset managed by the Foundation National Institute for Cancer Epidemiology and Registration (NICER).<br><br>http://www.nicer.org/en/statistics-atlas/	The Swiss national dataset managed by the Foundation National Institute for Cancer Epidemiology and Registration (NICER).<br><br>http://www.nicer.org/en/statistics-atlas/	United states CENSUS bureau<br><br>https://www.census.gov/en.html	93.4	2.0		1990-1994: 53.5% of data available 1995-1999: 56.9% of data available 2000-2004: 57.9% of data available 2005-2009: 61.8% of data available 2010-2014: 74.1% of data available
**France**	Data from health insurance administrative database:<br><br>Source Sniiram CNAMTS<br><br>Source INSEE, estimation of the population as of 1st January, published on 17th January 2017.	NA	Source INSEE, estimation of the population as of 1st January, published on 17th January 2017	>93.7	-	100%	All data available from 2007

**Latvia**	The Centre of Disease Prevention and Control, Republic of Latvia<br><br>Register for Patients with Particular Diseases, Patients with Cancer.	Register of Causes of Death	United states CENSUS bureau<br><br>https://www.census.gov/en.html	80.7	5.5	100%	-
**Catalonia**	El Càncer a Catalunya. Monografia 2016. Registre de cáncer de Catalunya. Pla Director d'Oncologia. http://cancer.gencat.cat/ca/professionals/estadistiques/<br><br>Registre del Càncer de Girona. Registre del càncer. Institut Català d'Oncologia http://ico.gencat.cat/ca/professionals/serveis_i_programes/registre_del_cancer/<br><br>Registre del Càncer de Tarragona. Funca - registre https://funca.cat/registre	El Càncer a Catalunya. Monografia 2016. Registre de cáncer de Catalunya. Pla Director d'Oncologia. http://cancer.gencat.cat/ca/professionals/estadistiques/<br><br>Registre de Mortalitat de Catalunya. Mortalitat. Departament de Salut http://salutweb.gencat.cat/ca/el_departament/estadistiques_sanitaries/dades_de_salut_i_serveis_sanitaris/mortalitat/	Institut d'Estadística de Catalunya. Idescat www.idescat.cat	91.7	2.2	100%	100%
**Slovenia**	Cancer Registry of Republic of Slovenia RS Data from: www.slora.si/en and information on: https://www.onko-i.si/eng/crs/	Cancer mortality data are collected by the National Institute of Public Health (NIPH)	United states CENSUS bureau<br><br>https://www.census.gov/en.html	95.2	<0.5	100%	-

**Portugal**	Registo Oncológico Regional do Norte	Registo Oncológico Regional do Norte	Registo Oncológico Regional do Norte	98.5%	-	100%	100%
**Czech Republic**	Czech National Cancer Registry	Czech National Cancer Registry and Czech Statistical Office (since 1994)	Czech Statistical Office	95.7	0.4	95%	100%
**United Kingdom**	Office for national statistics<br><br>https://www.ons.gov.uk/peoplepopulationandcommunity/healthandsocialcare/conditionsanddiseases/datasets/cancerregistrationstatisticscancerregistrationstatisticsengland	NA	United states CENSUS bureau<br><br>https://www.census.gov/en.html	84.6	0.1	98.4%	-
**Poland**	Krajowy Rejestr Nowotworów, Centrum Onkologii - Instytut im. Marii Skłodowskiej - Curie<br><br>http://onkologia.org.pl	Krajowy Rejestr Nowotworów, Centrum Onkologii - Instytut im. Marii Skłodowskiej - Curie<br><br>http://onkologia.org.pl	Krajowy Rejestr Nowotworów, Centrum Onkologii - Instytut im. Marii Skłodowskiej - Curie<br><br>http://onkologia.org.pl	>90.4	<2.9	100%	-

Supplementary table 1: Data source for the age-standardized incidence and mortality rates of colorectal cancer and population data. -: data was not available; * information retrieved from GLOBOCAN; º information retrieved from country specific database mentioned in column 2 (Incidence); NA = not applicable.

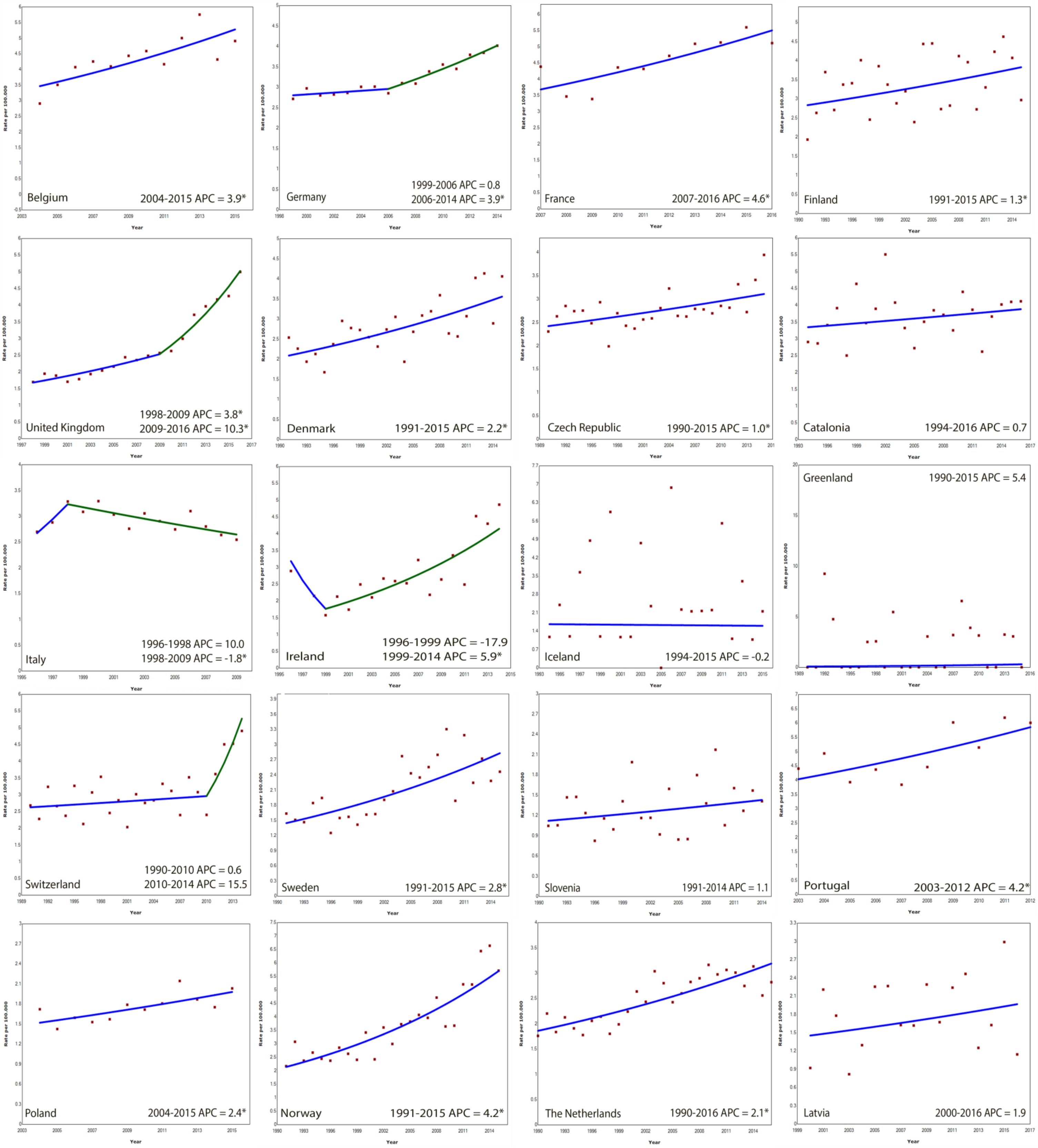

Supplementary figure 1: Incidence annual percent change (APC) per country in age group 20 to 39 year.

*Statistical significant change in trend.

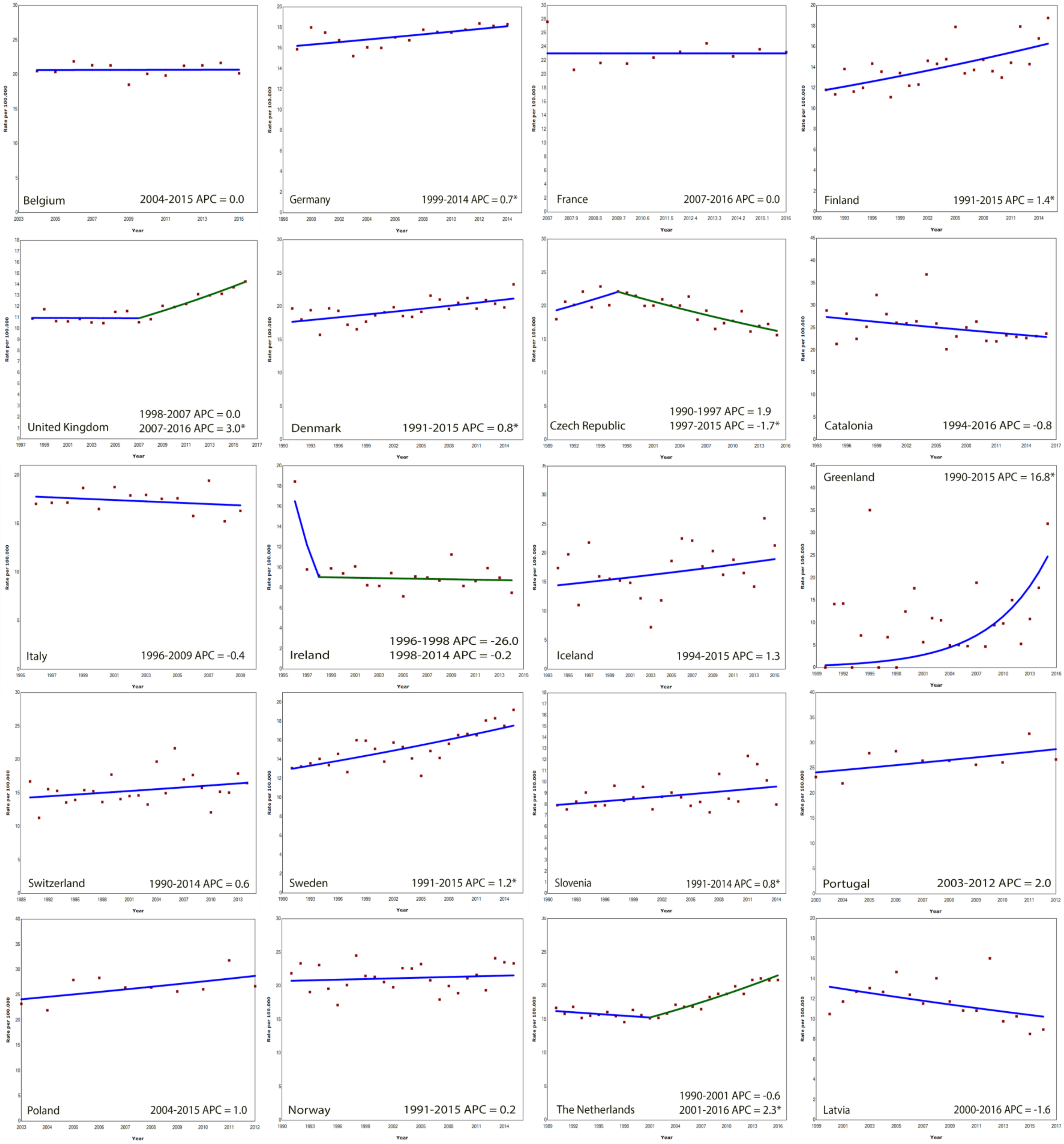

Supplementary figure 2: Incidence annual percent change (APC) per country in age group 40 to 49 year.

* Statistical significant change in trend.
